# A Method to Extract Causality for Safety Events in Chemical Accidents from Fault Trees and Accident Reports

**DOI:** 10.1155/2020/7132072

**Published:** 2020-06-19

**Authors:** Junwei Du, Hanrui Zhao, Yangyang Yu, Qiang Hu

**Affiliations:** School of Information Science and Technology, Qingdao University of Science and Technology, Qicngdao 266061, China

## Abstract

Chemical event evolutionary graph (CEEG) is an effective tool to perform safety analysis, early warning, and emergency disposal for chemical accidents. However, it is a complicated work to find causality among events in a CEEG. This paper presents a method to accurately extract event causality by using a neural network and structural analysis. First, we identify the events and their component elements from fault trees by natural language processing technology. Then, causality in accident events is divided into explicit causality and implicit causality. Explicit causality is obtained by analyzing the hierarchical structure relations of event nodes and the semantics of component logic gates in fault trees. By integrating internal structural features of events and semantic features of event sentences, we extract implicit causality by utilizing a bidirectional gated recurrent unit (BiGRU) neural network. An algorithm, named CEFTAR, is presented to extract causality for safety events in chemical accidents from fault trees and accident reports. Compared with the existing methods, experimental results show that our method has a higher accuracy and recall rate in extracting causality.

## 1. Introduction

In recent years, the chemical industry has made tremendous contributions to economic and social development. However, a series of safety accidents have occurred frequently along with the enormous economic benefits brought by chemical enterprises. For example, seventy-eight people died in the explosion of Yancheng Chemical Industrial Park on March 21, 2019. After the accident, sixteen chemical enterprises in this industrial park were closed [1]. The occurrence of chemical accidents has brought enormous economic losses to enterprises and individuals, made irreparable damage to the environment, and even caused heavy casualties [2]. Therefore, accident prevention and emergency treatment have become the focus of daily production in the chemical industry.

On the ascending scale of production in the chemical industry and abundance of chemical products, the production process is becoming more and more complex, and the risk factors in all aspects of production are also increasing [3]. Controlling and early warning the unsafe factors such as high temperature, high pressure, flammability, explosion, and poisoning in chemical production can effectively prevent future chemical accidents [4]. Versaci presented a fuzzy method to achieve the detection/classification of defects. It considered classes of defects to a certain depth characterized by typical ranges of fuzzy similarities [5]. The literature [6] covers the practical implementation of ultrasonic NDT techniques in an industrial environment, discussing several issues that may emerge and proposing strategies for addressing them successfully. Many of the technologies it provides can be applied to the detection of hazardous chemical production information.

By analyzing the causes of accidents, excavating the potential factors, evolution rules, and protective measures, we can decrease accident rates, reduce accident losses, and improve the safety management level and emergency disposal ability of chemical enterprises. Fault tree analysis is one of the most frequently used methods in safety analysis, prevention, and emergency disposal [7]. Fault trees can describe the causes of accidents and their temporal logic relationship [8]. So, we can find out the key events in chemical accidents and predict potential hazards in chemical production from the existing fault trees.

However, only the evolution process of an accident can be obtained from fault trees. Time, location, and environmental state of these accidents were not described for the concise structure of fault trees. Thus, it may result in a lack of important information while analyzing the cause of an accident. Since most of the fault trees were constructed based on expert's accident analysis experience, there may be semantic ambiguity, incomplete information, mixed information, and information hybridity in the fault trees. Meanwhile, for the complex fault trees, we may face high complexity and incomplete evolutionary information while analyzing the event evolution mechanism. It is also very difficult to accurately locate or match event evolution sequences.

To compensate for the abovementioned deficiencies of the fault trees in safety analysis, early warning, and emergency disposal, event evolutionary graph (EEG) is introduced to model the event evolution process in chemical accidents in this paper. EEG is a type of graphic information carrier developed on the basis of knowledge graphs [9]. Illustrated as a digraph, it describes the causal relationship and temporal dependence in the event chains of an accident. An EEG which described the evolution process of chemical accidents is called chemical event evolutionary graph (CEEG). By traversing the CEEG, we can easily obtain the evolution sequences of events in chemical accidents. We can also predict the potential events in an accident by means of evaluating the event causality and transfer probability.

**Figure 1:**
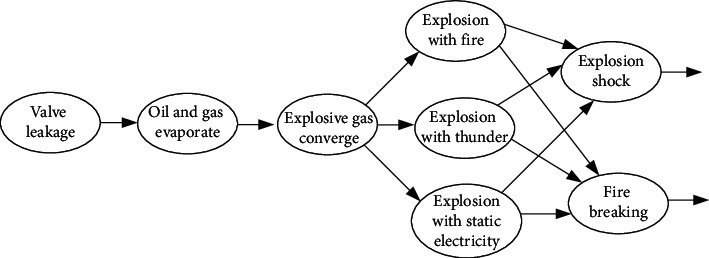
An event evolutionary graph under the scenario of the “volatile explosion of oil and gas.”

A scenario about the event of the “volatile explosion of oil and gas” is illustrated by the CEEG in Figure 1. The first node that is “valve leakage” says that the event starts from valve leakage of storage tanks. Then “oil and gas evaporate” and “explosive gas converge” occur sequentially. When the concentration of explosive gas exceeds a certain amount, it will cause an explosion. Explosion requires some triggering conditions. So, we can see that the node “explosive gas converge” connects with three succeeding nodes. “Explosion with fire,” “explosion with thunder,” and “explosion with static electricity” represent the explosion caused by the fire, thunder, and static electricity, respectively. “Explosion shock” and “fire breaking” are two main destruction scenarios. Thus, the nodes “explosion shock” and “fire breaking” are linked with three types of explosion nodes separately. Since the events in an accident are organized by their temporal or causal relationships, we can easily achieve the event traceability and early warning with a CEEG.

It is a complicated and challenging task to build the CEEG. Event identification, event relation extraction, and event entity link are the main tasks in the process of constructing the EEG. In this study, we build CEEG based on the existing fault trees and accident reports. Most events in the chemical accidents are with the causal relationship, and the causality is also the main link relation between safety events in the CEEG. So, we concern about how to identify events and extract causal relationships between these events. The main contributions of this study are as follows:

1. We propose an effective method to extract event elements by combining fault tree with accident reports. The combination of fault tree and accident report greatly reduces the complexity of event extraction based on NLP.
2. We obtain explicit causality by analyzing hierarchical structure relations of event nodes and logic gates in fault trees. Implicit causality is generated based on BiGRU neural network by feeding internal structural features of events and semantic features of event sentences. The accuracy and efficiency of extracting causality are improved by dividing causality into explicit causality and implicit causality.
3. We have conducted several rounds of experiments to verify the effectiveness of the proposed method. In view of accuracy and recall rate, experimental results show that our model and method are superior to the state-of-the-art methods in extracting causality.

The rest of this paper is structured as follows. In Section 2, we introduce the formal definitions of fault tree and EEG.  Section 3 presents a method to achieve event identification. How to extract causality between safety events is elaborated in Section 4. In Section 5, experiments are presented to show the effectiveness of our method. We conclude our work in Section 6.

## 2. Related Definitions

The main purpose of this paper is to provide an effective method of finding potential events and their causality. After getting events and their causality, we can build CEEG and then apply accident analysis, reasoning, and early warning. To accurately and automatically acquire the knowledge in building CEEG, we proposed a method to extract the events and causality from fault trees and accident reports. We will present the definitions of fault tree and EEG so as to better illustrate our method in the following sections.

Fault trees are frequently used to analyze the risks related to safety and they can describe the temporal logic of the events involved in a safety accident [10]. There are two types of nodes: events and gates in a fault tree. Events in a fault tree are used to represent the main events leading to accidents and they can be classified into three types: basic events, intermediate events, and top events. Gates represent how events propagate through the system while the edges were employed to express the occurring order relations of these events [11].

**Figure 2:**
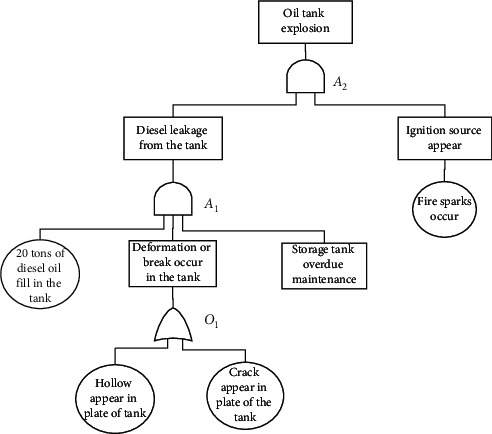
A fault tree under the scenario of “oil tank explosion.”

The fault tree in Figure 2 described a scenario of an “oil tank explosion.” We can see that the basic events “hollow appeared in plate of the tank” and “crack appeared in plate of the tank” are connected with the OR gate *O*_1_. It means that the event “deformation or break occurred in the tank” will be triggered if one of the above basic events has happened. For the AND gate *A*_1_, the events “20 Tons diesel oil filled in the tank,” “deformation or break occurred in the tank,” and “storage tank overdue maintenance” are its input events, and “diesel leakage from the tank” is its output event. So, only all the input events appear simultaneously, and the output event can occur. Similarly, we can deduce the sequence of events for “fire sparks occur,” “ignition source appear,” and “oil tank explosion.”

### 2.1. Definition 1 (Fault Tree)

A fault tree is a 4-tuple *FT* = (*V*, *G*, *E*, *v*_0_), consisting of the following components:

1. *V* is the set of nodes in *FT*; each node *v* in *V* is used to represent an event
2. *G* is the set of logic gates. ∀*g* ∈ *G*, *T* (*g*) is a function that describes the type of each gate
3. *E* is the set of arcs in *FT*, *E* ⊆ *V* × *G* ∪ *G* × *V*
4. *v*_0_ is the root node of *FT*

There are three types of nodes in fault trees: root node, leaf nodes, and intermediate nodes. Root node *v*_0_ represented the top event. *V*_L_ = {*v* ∈ *V* ∧ (∄*g* ∈ *G*, s.t. (*v*, *g*) ∈ *E*)}; ∀*v* ∈ *V*_L_, *v* is a leaf node, and it is used to denote a basic event. *V*_M_ = {*v* ∈ *V* ∧ (∃*g* ∈ *G*, s.t. (*v*, *g*) ∈ *E*)}; ∀*v* ∈ *V*_M_, *v* is an intermediate node, and it is used to denote an intermediate event.

To easily obtain the input events and output event for a logic gate, we present two functions: (1) *I*: *G* ⟶ Ψ (*E*) describes the input event of each gate; (2) *O* : *G* ⟶ Γ (*E*) describes the output event of each gate.

From the example in Figure 1, we can see that an event evolutionary graph is a digraph. Nodes in event evolutionary graph are used to denote the events, and the arcs are adopted to represent the dependencies among these events. Now, we give the definition of EEG.

### 2.2. Definition 2 (Event Evolutionary Graph)

Event evolutionary graph (*EEG*) = (*V*, *E*). Here, *V* is a set of nodes; ∀*v*_*i*_ ∈ *V*, *v*_*i*_ is an event, and it is represented by abstract, generalized, and semantic complete verb phrase. *E* is a set of arcs; ∀*e*_*ij*_ ∈ *E*, it denotes that there exists dependency between the event *v*_*i*_ and *v*_*j*_.

There are two kinds of dependencies between the events: sequential relation and causality. The sequential relation between two events refers to their partial temporal orderings. Causality is the relation between one event (the cause) and a second event (the effect), where the second event is understood as a consequence of the first [9]. In this study, we used the symbol “⟶” to represent causality. For two events *e*_*i*_ and *e*_*j*_, *e*_*i*_ ⟶ *e*_*j*_ means that *e*_*i*_ is the cause of *e*_*j*_. It is obvious that the causal relation between events must be sequential. To find causality between two events is a more difficult and challenging work.

## 3. Event Identification

Event identification, also called event recognition or event extraction, is the process to find the component elements (factors) of an event from various information sources. In a recent study, Skarlatidis addressed the issue of uncertainty in logic-based event recognition by extending the Event Calculus with probabilistic reasoning [12]. Chen introduced a word-representation model to capture meaningful semantic regularities for words. He adopted a framework based on a dynamic multipooling convolutional neural network (DMCNN) to capture sentence-level clues and reserve crucial information [13]. Feng developed a language-independent neural network to capture both sequence and chunk information from specific contexts and used them to train an event detector for multiple languages without any manually encoded features [14]. Liao proposed a new event recognition method based on positive and negative weighting proposed by constructing a trigger table [15]. Hogenboom gave a summarization of event extraction techniques for textual data, distinguishing between data-driven, knowledge-driven, and hybrid methods, and presented a qualitative evaluation of these methods [16].

In this study, we will extract events and investigated their causal relationship in chemical accidents. The information source of our event identification is fault trees and accident reports. Now, we give the formal structure of the event used in this paper.

### 3.1. Definition 3 (Event)

An event in an accident is formally defined as a 4-tuple *e* = {*o*, *v*, *p*, *t*}, where *o*, *v*, *p*, and *t* are used to represent the event participants, event trigger word, location, and timestamp of event occurrence, respectively.

To concisely demonstrate an evolutionary process, fault trees were normally designed with summary information of events. We cannot find a detailed description of the information about the time, location, and environment state. Such information is elaborated in the accident reports. So, we can acquire these event elements by the natural language processing technology from fault trees and accident reports. The extraction of event elements includes the following work: corpus segmentation, part-of-speech tagging, semantic role labeling (SRL), semantic dependency parsing (SDP), and dependency parsing (DP) [17,18]. For each node in a fault tree, we can obtain event elements by the following steps:

1. Participant ⟵ SRL (fault tree node)
2. Trigger-word ⟵ SRL (fault tree node)
3. Place ⟵ SDP (event sentences) and (Place.semantic-dependency (Trigger-word) = LOC)
4. Time ⟵ SDP (event sentences) and (Time.semantic-dependency (Trigger-word) = Time)
5. Subject ⟵ DP (event sentences) and (Subject.dependency-parsing (Trigger-word) = SBV)
6. Object ⟵ DP(event sentences) and (Object.dependency-parsing (Trigger-word) = VOB)

SRL is first used to identify the trigger words and participants of events in a fault tree. Timestamp and position for an event can be obtained by SDP technology from trigger words. The whole information about the event will be generated after the “subject-predicate-object” structure was parsed by DP. The aforementioned processing functions (SRL, SDP, and DP) were normally encapsulated as APP services. Here, the open-sourced natural language processing system developed in the Research Center for Social Computing and Information Retrieval of Harbin Institute of Technology was invoked in our study to parse event sentences [19].

**Figure 3:**
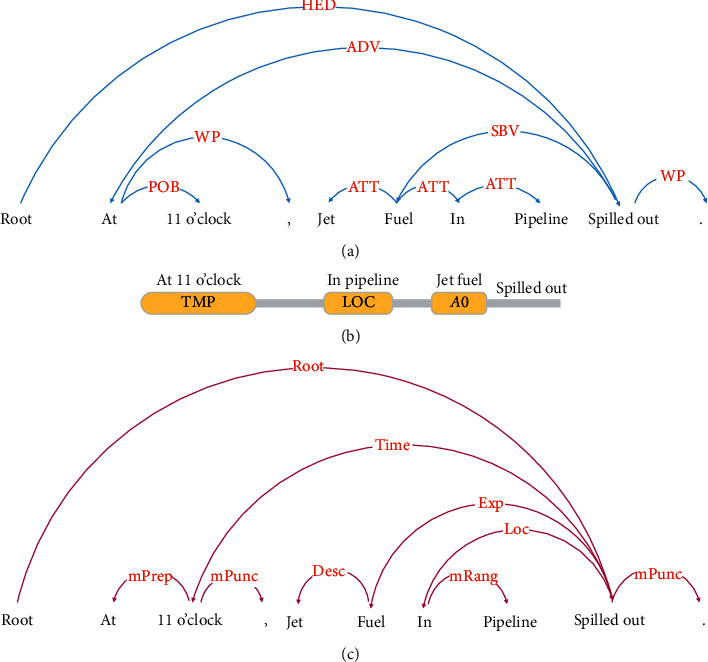
An example of event identification.

In Figure 2, there is a node labeling “jet fuel spilled out” in a fault tree. The event sentence of this node in the corresponding accident report is “At 11 o'clock, jet fuel in pipeline spilled out.” The processing results of SDP, SRL, and DP are shown in (a), (b), and (c) of Figure 3. We can see that “jet fuel” is the event participant while “spilled out” is an event trigger word.

SDP can identify semantic roles and their relationships in event sentences. The main relations between different roles include the agent relationship, the patient relationship, and the experiencer relationship. The result of SDP in Figure 3(b) shows that the participant “jet fuel” and trigger word “spilled out” are with the experiencer relationship. “In pipeline” and “at 11 o'clock” are of semantic dependence with a trigger word. The roles of “in pipeline” and “at 11 o'clock” were position and time, respectively. Therefore, the participant in this sentence is “jet fuel,” the trigger word is “spilled out,” the occurrence time is “at 11 o'clock,” and the place of occurrence is “in pipeline.” The relations of different words in the sentence were illustrated in Figure 3(c) by DP. So far, we can get all event elements of the sentence and the 4-tuple *e* = {jet fuel, spilled out, in pipeline, at 11 o'clock}.

## 4. Extraction of Event Causality

A fault tree is a kind of logical causality digraph including the symbols of events, logic gates, and transitions. It can show the variety of system states by the logical evolution of basic events. Event causality in a fault tree can be divided into two categories: explicit causality and implicit causality.

### 4.1. Extraction of Explicit Causality

Explicit causality can be extracted by analyzing the hierarchical structure relations of event nodes and the semantics of component logic gates. There are various types of logic gates in fault trees. Normally, the following three types of logic gates, namely, AND gate, OR gate, and VOT (*k*/*N*) gate, are the fundamental gates. By the combination of the above logic gates, we can get the semantic logic of all the other gates used in fault tree [11].

Let *F* be a fault tree and let *BE* represent the set of basic events in *F*. The semantics of *F* is a function *π*_*F*_: Ψ (*BE*) × *E* ⟶ {0,1} where *π*_*F*_ (*S*, *e*) indicates whether *e* fails given the set *S* of failed *BE*. It is defined as follows:

1. For *e* ∈ *BE*, *π*_*F*_ (*S*, *e*) = *e* ∈ *S*
2. For *g* ∈ *G* and *T* (*g*) = AND, let *π*_*F*_ (*S*, *g*) = $\underset{x\in I\left(g\right)}{\wedge }\pi_{F}\left(S,x\right)$
3. For *g* ∈ *G* and *T* (*g*) = OR, let *π*_*F*_ (*S*, *g*) = $\underset{x\in I\left(g\right)}{\vee }\pi_{F}\left(S,x\right)$
4. For *g* ∈ *G* and *T* (*g*) = VOT (*k*/*N*), let *π*_*F*_ (*S*, *g*) = $\underset{x\in I\left(g\right)}{\Sigma }\pi_{F}\left(S,x\right)\geq k$

**Figure 4:**
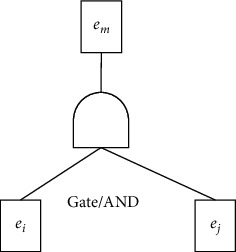
An example of a basic structure with AND gate.

From the semantics of logic gates, we know that events in lower-level nodes are the cause of events in upper-level nodes.  Figure 4 illustrates a basic structure in a fault tree. Two events *e*_*i*_ and *e*_*j*_ were connected by the logic gate AND, and the event *e*_*m*_ is located in the upper-level node. So, we can get two explicit causality rules: *e*_*i*_ ⟶ *e*_*m*_ and *e*_*j*_ ⟶ *e*_*m*_. For a given fault tree, we can obtain the explicit causality rules by traversing all the logic gates.

### 4.2. Extraction of Implicit Causality

Explicit causality can be easily discriminated from the hierarchical structure of event nodes in fault trees. However, there may be some hybrid information in the event nodes. Meanwhile, multiple events were occasionally described in one event node. Thus, some implicit causality may be hidden in the events of fault trees. Implicit causality should be extracted so as to build a correct CEEG. There are two steps in finding implicit causality. One is to investigate whether there is a causal relationship between two events and the other is to determine causal direction. The causal direction is used to describe which event is a cause and which one is the result. In this study, every two events in the fault tree nodes were assembled as candidate event pairs. By analyzing the internal structure of the events and semantic features of event sentences, we can identify the causal relationship and its direction with the help of our causal classifier.

Liu proposed an experience-based causality learning framework. Compared to traditional approaches, which attempt to handle the causality problem relying on textual clues and linguistic resources, they are the first to use experience information for causality learning [20]. Riaz focused on identifying and employing semantic classes of nouns and verbs with a high tendency to encode cause or noncause relations [21]. Zhao designed an abstract causality network and a dual cause-effect transition model. It is effective for discovering high-level causality rules behind specific causal events [22]. Zhao and Liu presented a new Restricted Hidden Naive Bayes model to extract causality from texts. It can cope with partial interactions among features so as to avoid overfitting problems on the Hidden Naive Bayes model, especially the interaction between the connective category and the syntactic structure of sentences [23]. A framework that combines intuitionistic fuzzy set theory and expert elicitation was proposed to enable quantitative analysis of temporal fault trees of dynamic systems with uncertain data [24].

In recent years, various types of neural networks and deep learning models have provided favorable support for the popularization and application of machine learning. For example, Deng proposed an improved quantum-inspired differential evolution method to construct an optimal deep belief network, which is further applied to propose a new fault classification [25]. An improved ant colony optimization algorithm based on the multipopulation strategy, coevolution mechanism, pheromone updating strategy, and pheromone diffusion mechanism is proposed to balance the convergence speed and solution diversity and improve optimization performance in solving large-scale optimization problem [26]. Similar work about improved coevolution ant colony optimization algorithm with Multistrategies is presented in the literature [27]. Zhao extended a broad learning system based on the semisupervised learning of manifold regularization framework to propose a semisupervised broad learning system. It can achieve higher classification accuracy for different complex data and takes on fast operation speed and strong generalization ability [28]. These methods are of great significance for us to mine and optimize causality by using neural networks.

In this study, we present a new method to obtain implicit causality by transforming the causality extraction into a binary classification problem. Four steps including internal structural features extraction of events, semantic features extraction of event sentences, feature fusion, and softmax classification were adopted to find implicit causality in a fault tree.

**Figure 5:**
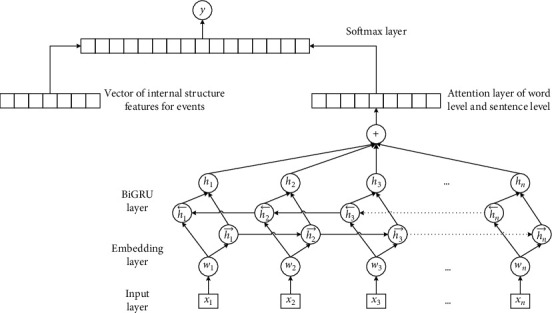
Extraction process of implicit causality.

As shown in Figure 5, word (term) vector is first employed to express the lexical sequence feature of the event sentence. Then, BiGRU neural network is used to mine the context semantic features of the event sentence. To improve the accuracy of context semantic, we add the attention mechanism into the BiGRU model at the level of word and sentence. Finally, both semantic features and internal structure characteristics are input into softmax classifier to determine whether there are a causal relationship and the causal relationship direction between the given events.

#### 4.2.1. Extraction of Internal Structure Features for Events

Internal structure features of events refer to the relationship characteristic of component elements in event pairs. Let *e*_*i*_ = {*o*_*i*_,*v*_*i*_*, p*_*i*_*, t*_*i*_} be an event, where 0 ≤ *i* < = *n*. *E* = {*e*_*i*_} is a set of events. ∀*e*_*i*_ and *e*_*j*_ ∈ *E*, <*e*_*i*_, *e*_*j*_> can form an event pair. Three internal structure features of event pairs were investigated in this section:

1. Appearing probability*: P* (*e*_*i*_) was employed to represent the appearing probability of *e*_i_. *Pc* (*e*_*i*_, *e*_*j*_) is defined as the cooccurrence probability of *e*_*i*_ and *e*_*j*_. Furthermore, *Pc* (*e*_*i*_ ⟶ *e*_*i*_) is the cooccurrence probability of *e*_*i*_ and *e*_*j*_ with the condition that *e*_*i*_ is the cause while *e*_*j*_ is the result. For the event elements, we present a group of appearing probability. *P*(*e*_i_.*o*) is used to express the appearing probability participant *e*_*i*_.*o*. Similarly, *P* (*e*_*i*_.*v*), *P* (*e*_*i*_.*p*), and *P* (*e*_*i*_.*t*) are the appearing probability of trigger word, location, and timestamp of event, respectively.
2. Pointwise mutual information: pointwise mutual information (PMI) is usually used to calculate the semantic similarity between two words [29]. The basic idea for PMI is to count the probability of two words simultaneously appearing in the text. Normally, two words are concluded with a high correlation for their higher PMI. Thus, PMI of events and their elements can be used to determine the correlation degree between two events. Definition of PMI for the event pairs and event elements can refer, respectively, to

(1) $$\begin{array}{c} \text{PMI}\left(e_{i},e_{j}\right)=\log\frac{P\left(e_{i},e_{j}\right)}{P\left(e_{i}\right)*P\left(e_{j}\right)}, \end{array}$$

(2) $$\begin{array}{c} \text{PMI}\left(e_{i}.f,e_{j}.f\right)=\log\frac{P\left(e_{i}.f,e_{j}.f\right)}{P\left(e_{i}.f\right)*P\left(e_{j}.f\right)},\;f\in \left\{o,v,p,t\right\}. \end{array}$$

(3) Position relevancy between events: events contained in fault tree nodes may exist in different sentences. Two sentences are generally considered with more dependence or causality if they are located closely. The distance between sentences is inversely proportional to the degree of relationship between the sentences. Paragraph sentences containing events are numbered sequentially from zero. Let *TS* be the total number of sentences in an accident report. *SP* (*e*_*i*_) is used to represent the number of the sentence including *e*_i_. Relative position for an event pair <*e*_*i*_, *e*_*j*_>, namely, *SPe*_*ij*_, is assigned as *SP* (*e*_*i*_)−*SP* (*e*_*j*_). Position relevancy is defined as *PRe*_*ij*_, *PRe*_*ij*_ = 1−*SPe*_*ij*_/*TS.*

We build a 19-v vector *ISFe*_*ij*_ to express the internal structure features for event pair <*e*_*i*_, *e*_*j*_>. Here, *ISFe*_*ij*_ = (*P* (*e*_*i*_), *P* (*e*_*j*_), *P* (*e*_*i*_.*o*), *P* (*e*_*j*_.*o*), *P* (*e*_*i*_.*v*), *P* (*e*_*j*_.*v*), *P* (*e*_*i*_.*p*), *P* (*e*_*j*_.*p*), *P* (*e*_*i*_.*t*), *P* (*e*_*j*_.*t*), *Pc* (*e*_*i*_, *e*_j_), *Pc* (*e*_*i*_ ⟶ *e*_*j*_), *Pc* (*e*_*j*_ ⟶ *e*_*i*_), *PMI* (*e*_*i*_.*o*, *e*_*j*_.*o*), *PMI* (*e*_*i*_.*v*, *e*_*j*_.*v*), *PMI* (*e*_*i*_.*p*, *e*_*j*_.*p*), *PMI* (*e*_*i*_.*t*, *e*_*j*_.*t*), *PMI* (*e*_*i*_, *e*_*j*_), *PRe*_*ij*_).

#### 4.2.2. Extraction Semantic Feature in Event Sentences

*(1) BiGRU Neural Network*. Semantic dependence of two events can be obtained from event sentences. Semantic features of event sentences were taken as one of the features to identify event relations in our study. The tool “Word2vec” was used to train word embedding for the terms in the corpus of chemical accidents [30]. Then, event sentences can be expressed by the word embedding sequences. The word vectors were derived from the text training set of accident reports and some Internet accident news after denoising. Given a sentence consisting of *n* words, every word *w* is represented by a real-valued vector, and the vector of the sentence is represented as *S* = (*w*_1_, *w*_2_,…, *w*_*n*_).

GRU neural network is a popular variant of the LSTM neural network. Compared with LSTM, GRU is with a more succinct structure [31]. GRU has only two control gates: update gate and reset gate. The information dissemination in GRU can be described as follows:

1. Update gate: the update gate *z*_*t*_ (see formula (3)) is used to control the extent to which the state information of the previous moment is brought into the current state. The larger the value of the update gate is, the more the state information of the previous moment can be brought in:

(3) $$\begin{array}{c} z_{t}=\sigma \left(W_{z}*\left[h_{t-1},x_{t}\right]\right). \end{array}$$

(2) Reset gate: reset gate *r*_*t*_ (see formula (4)) is used to control the degree of ignoring the state information of the previous moment. The smaller the value of reset gates is, the more the state information of the preceding moment is ignored:

(4) $$\begin{array}{c} r_{t}=\sigma \left(W_{r}*\left[h_{t-1},x_{t}\right]\right). \end{array}$$

Get a new hidden state; *z*_*t*_ and *r*_*t*_ jointly controlled how to obtain new hidden state *h*_*t*−1_from the previously hidden state *h*_*t*_ as follows:

(5) $$\begin{array}{c} \tilde{h_{t}}=\tan h\left(W*\left[r_{t}*h_{t-1},x_{t}\right]\right), \end{array}$$

(6) $$\begin{array}{c} h_{t}=\left(1-z_{t}\right)*h_{t-1}+z_{t}*\tilde{h_{t}}. \end{array}$$

Compared with LSTM, GRU has the advantages of simple structure, fewer parameters, and fast training speed. It has shown a superior performance than LSTM. We use the accident text set to train the neural network. Event sentences in accident reports were first obtained according to the fault tree. Vectors of these event sentences were then input to the neural network to extract the semantic features of event sentences.

One-way neural network propagates from front to back, which can only contain the transmission of the previous information. The reverse transmission of the latter information cannot be propagated. The bidirectional neural network consists of two neural networks to train sequence forward and backward, respectively, and outputs two result sequences containing complete context information [32]. Here, we use the element-wise sum to combine the forward and backward pass outputs:

(7) $$\begin{array}{c} \overset{⟶}{h_{t}}=\overset{⟶}{\text{GRU}}\left(wt,\overset{⟶}{h_{t-1}}\right), \end{array}$$

(8) $$\begin{array}{c} \overset{⟵}{h_{t}}=\overset{⟶}{\text{GRU}}\left(wt,\overset{⟵}{h_{t-1}}\right), \end{array}$$

(9) $$\begin{array}{c} H=\overset{⟶}{h_{t}}⊕\overset{⟵}{h_{t}}. \end{array}$$

*(2) Attention Mechanism*. Attentive neural networks have recently demonstrated great success in a wide range of tasks, such as question answering, machine translations, and image recognition. We can apply attention computation for any two words in a sentence by introducing a self-attention mechanism. Thus, the dependence relationships of words in sentences can be learned more precisely. Word-level attention mechanism proposed by Zhou et al. [33] and sentence-level attention mechanism proposed by Lin et al. [34] for text representation have been widely concerned. In this section, we combine the above two methods to generate vectors for sentences.

In general, for an event pair <*e*_*p*_, *e*_*q*_>, *e*_*p*_ and *e*_*q*_ were located in different sentences. Assume that there are *L* sentences between the event *e*_*p*_ and *e*_*q*_. The L sentences form a set *Se*_*pq*_. Given a sentence *Se*_*i*_ in *Se*_*pq*_, *T*_*i*_ was the number of words in sentence *Se*_*i*_. *w*_*it*_ with *t*∈ [1, *T*_i_] represents the *t*^th^ word in *Se*_*i*_. We obtain an annotation for a given word *w*_*it*_ by concatenating the forward hidden state and backward hidden state$hit=\overset{⟶}{hit}⊕\overset{⟵}{hit}$. Once every word is assigned with weight, we can give an annotation for the sentence.

An activation function tan *h*(*x*) in formula (10) was used to handle hit. Then, we measure the importance of the word with a trained parameter vector *W*_1_ and get a normalized importance weight *α*_*it*_ through a softmax function. Sentence vector *S*_*i*_ can be obtained by using a weighted sum of all the word annotations with their weight by the following:

(10) $$\begin{array}{c} \alpha_{it}=\frac{\exp\left(W_{1}^{T}*\;\tan h\left(h_{it}\right)\right)}{\sum_{t}\exp\left(W_{1}^{T}*\;\tan h\left(h_{it}\right)\right)}, \end{array}$$

(11) $$\begin{array}{c} s_{i}=\sum_{t}\alpha_{it}*h_{it}. \end{array}$$

Here, *h*_*it*_ ∈ *R*^d^W^*∗t*^, d^w^ is the dimension of the word vectors, *W*_1_ is a trained parameter vector, and *w*_*i*_^*T*^ is a transpose, *W*_1_ ∈ *R*^1*∗d*^*W*^^, and *s*_*i*_ ∈ *R*^d^W^*∗*1^.

We first feed the word annotation of *S*_*i*_ into a one-layer MLP so as to get *u*_*i*_ as a hidden representation of *S*_*i*_. Formula (13) was adopted to compute the weight of a sentence. We compute the vector *v*_*s*_ for *Se*_*pq*_ that summarizes all the information of sentences containing the event pairs:

(12) $$\begin{array}{c} \mu_{i}=\tan h\left(W_{s}*s_{i}+b_{s}\right), \end{array}$$

(13) $$\begin{array}{c} \alpha_{i}=\frac{\exp\left(u_{i}*r\right)}{\sum_{i}\exp\left(u_{i}*r\right)}, \end{array}$$

(14) $$\begin{array}{c} v_{s}=\sum_{i}\alpha i*s_{i}. \end{array}$$

*(3) Layer Normalization*. During the training process of a deep learning network, parameter changes will lead to the distribution variation of input data in the subsequent network. To solve the problem of data distribution variation in the training process of the middle layers, Ioffe proposed the BN algorithm [35]. For each batch, the sum input distribution is used to calculate the mean and variance, which are used to normalize the sum input of neuron in each training sample. This method significantly reduces the training time of the precursor neural network. However, the effect of batch normalization depends on the size of minibatch. It is necessary to count the first-order and second-order statistics of each minibatch in the running process, which cannot be widely used in RNN networks. Therefore, Ba et al. proposed the concept of layer normalization (LN), which reduces training time by calculating the mean and variance of the sum input on one-layer neurons [36]:

(15) $$\begin{array}{c} h_{t}=f\left[\frac{g}{\sigma^{t}+\zeta }⊙\left(a^{t}-\mu^{t}\right)+b\right]. \end{array}$$

Here, at is the input parameters of each layer, *μ*^*t*^ is the average value of input data, and *σ*^*t*^ is the input variance. *g* and *b* are bias constants, *f* is a linear transformation, and *ζ* is a regularization parameter. In this study, the LN method was introduced into formulas (4)–(6) to improve the training speed of the GRU neural network.

#### 4.2.3. Fusion of Features and Classification for Events

We have presented a method to obtain the internal structure features for events and semantic features in event sentences. In this section, we achieve the fusion of features and classification of causality.

There are three kinds of classification results for softmax classifier, which indicate whether two events have causality and causality direction. *v*_*s*_ is a sentence vector obtained from formula (14), *v*_*e*_ is the vector of event structure feature, and *W*_*f*_ is the model training parameter. *y* (see formula (16)) is used to express the classification result of two types of feature fusion:

(16) $$\begin{array}{c} y=\arg\;\max\left(\text{soft}\max\left(W_{f}*\left(v_{s}+v_{e}\right)\right)\right). \end{array}$$

Meanwhile, cross-entropy was introduced to serve as a training objective function (see formula (17)). In formula (17), *n* is the number of sentences and *θ* represents all the parameters in the model:

(17) $$\begin{array}{c} H\left(\theta \right)=\sum_{i=1}^{n}\log\;p\left(\left.r_{i}\right|s_{i},\theta \right). \end{array}$$

### 4.3. Algorithm for Causality Extraction

In this section, we summarize the main operating steps of our proposed method. An algorithm, namely, CEFTAR, is presented to extract causality from fault trees and chemical accident reports.

In te Algorithm 1, we first construct three sets. They are the set of events (ES), event pairs (EPS), and event pairs with causality (ECS). All these sets are initialized as empty sets. From line (3) to line (4), we use the popular word segmentation tool “Jieba” to obtain all the words in the chemical accident reports. So, we can get a corpus based on these words. Meanwhile, the tool “Word2vec” is employed to generate vectors for the words in the corpus. By traversing all the fault trees in FTS, we can add all the events into the ES (see line (5) to line (8)). In line (9), event pairs are generated with any combination of events in ES. All the event pairs are added to EPS.

For an event pair, we first extract explicit causality (see line (11) to line (12)). If two events are located in different hierarchical structures and connected with the same logic gate, they have explicit causality. Implicit causality will be further investigated once they are not with explicit causality. After analyzing the internal structural feature for the event pair, we construct *ISFe*_*ij*_ and use *v*_*e*_ to represent the vector of *ISFe*_*ij*_. Then, semantic features of sentences including the even pair are obtained by the following steps. We get all the sentences between the two events and compute the vector for these sentences based on BiGRU neural network. Finally, the combination vector of the internal structural feature and the sentence semantic feature is sent to a softmax classifier to decide whether the two events have implicit causality (see line (13) to line (18)). ECS is returned by the CEFTAR algorithm as the final result of causality. The meanings of parameters in all the formulas and symbol abbreviations are presented in Table 1.

## 5. Experiment and Analysis

In this section, we present experiments to validate the effectiveness of the proposed model and method. Our experiments were performed on the dataset which consists of 5867 accident reports and fault trees. Five experts in the domain of chemical accident analysis were employed to extract and annotate the causality in these reports and fault trees.

The hardware of the computer is as follows: CPU is i7-8700 with 3.2 GHz, six cores, and twelve threads. The memory is 16G. The Graphics card is GTX1060 with 6G. Tensorflow was adopted to implement the causal relationship extraction model in this study. Five rounds of experiments were performed and the average values were taken as the experimental results. A grid search algorithm is used to test the combination of different parameters to determine the optimal parameters for our model. The values of optimal parameters in our model are shown in Table 2.

**Figure 6:**
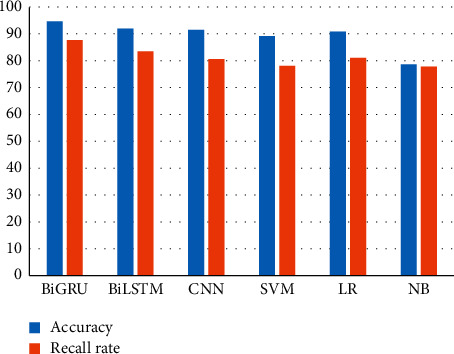
Accuracy and recall rate for different models.

We compared our model with other frequently used machine learning or neural network models to show its advantages. From Figure 6, we can see that our model is with higher accuracy and recall rate in extracting causality than BiLSTM, CNN, SVM, LR, and NB. We can see that the accuracy and recall rate of BiGRU, BiLSTM, and CNN are higher than those of SVM, LR, and NB. It is because the neural network model is superior to the traditional machine learning model for mining the hidden features. BiGRU and BiLSTM had higher accuracy and recall rate than CNN since LSTM networks can better capture context features for long text sequences, while CNN is suitable for capturing local features.

**Figure 7:**
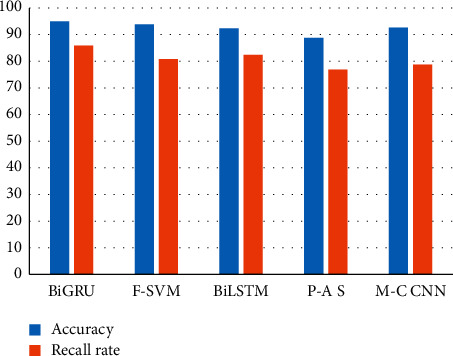
Accuracy and recall rate for different methods.

Four state-of-the-art methods including Feature-SVM (F-SVM) [8], BiLSTM [37], pattern-argument semantics (P-A S) [38], and Multicolumn CNN (MCCNN) [39] were also executed on the same dataset to obtain causality. As shown in Figure 7, the accuracy and recall rate of our method is the highest one. Thus, experimental results show that our proposed model and method in extracting causality are superior to the existing methods.

**Figure 8:**
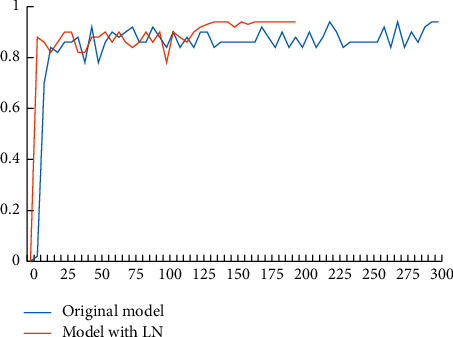
The effect of LN layer normalization on model performance.

Two data curves are shown in Figure 8, in which the abscissa is the number of running steps and the ordinate is the model accuracy. We can see that the LN layer normalization accelerated network convergence and reduced operation time and cost.

## 6. Conclusions

CEEG is an EEG describing the evolution process of chemical accidents. We can easily obtain evolution sequences of events in chemical accidents. Safety analysis, early warning, and emergency disposal can be performed based on these evolution sequences. To accurately and easily obtain the causality in building CEEG, a method to extract causality for safety events in chemical accidents from fault trees and accident reports is proposed in this paper.

We propose an effective method to extract events and their elements by combining fault tree with accident reports. Causality between these events is divided into explicit causality and implicit causality. We obtain explicit causality by analyzing hierarchical structure relations of event nodes and logic gates in fault trees. Implicit causality is generated based on BiGRU neural network by feeding internal structural features of events and semantic features of event sentences. Experimental results show that the proposed method conduces to better performance in accuracy and recall rate during the process of extracting causality.

In future work, more elements of events affecting chemical accidents will be taken into consideration, such as the environment, weather, and policy guidance factors. The accuracy will be further increased after more elements are adopted to model the events. Meanwhile, more cases of chemical accidents will be collected so as to enrich the training dataset. The proposed method will get better performance after adjusting the optimal model parameters with more abundant data available.

## Figures and Tables

**Algorithm 1:**
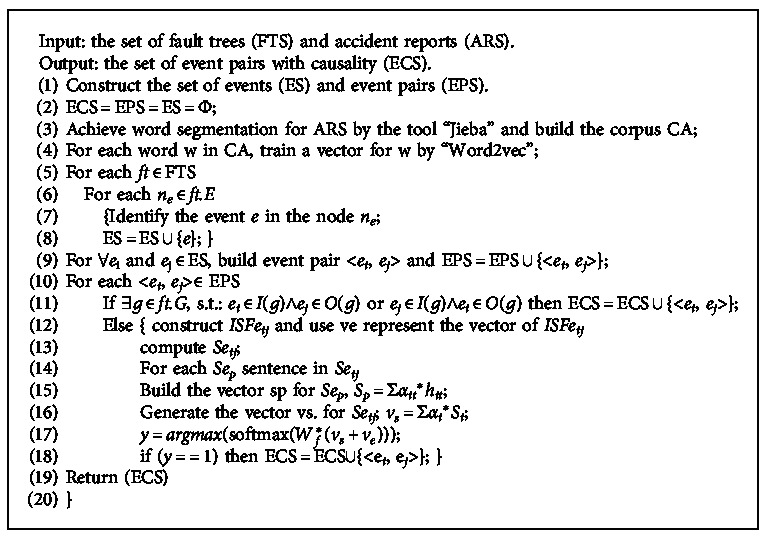
The CEFTAR algorithm.

**Table 1:** Notations and meanings.

Notation	Meaning
*FT (V, G, E, v*_*0*_)	Fault tree, where *V* is the set of nodes, *G* is the set of gates, *E* is the set of edges, and *v*_0_ is the root node
*V*_*M*_, *V*_*L*_	Set of intermediate nodes and set of leaf nodes
*EEG* = (*V*, *E*)	The expression of event evolutionary graph, where *V* is the set of nodes and *E* is the set of edges
Ψ(.)	The function that returns the input events for a given logic gate
Γ(.)	The function that returns the output event for a given logic gate
*e* = {*o*, *v*, *p*, *t*}	Event *e*, where *o*, *v*, *p*, and *t* are used to represent the event participants, event trigger word, location, and timestamp of event occurrence, respectively
*SRL*(.)	Semantic role labeling function
*SDP*(.)	Semantic dependency parsing function
*DP*(.)	Dependency parsing function
*π*_*F*_(*S*, *e*)	The function to judge whether *e* fails given the set *S* of failed *BE*
*P*(.)	Probability function
*Pc*(*., .*)	The cooccurrence probability function
*PMI*	Pointwise mutual information
*z*_t_	The update gate of GRU unit
*r*_t_	The reset gate of GRU unit
*x*_t_	The input of GRU unit
*h*_t_	The hidden layer information at the current moment
*h*_t-1_	The hidden layer information at the previous moment
${\tilde{h}}_{t}$	The candidate hidden layer information at the current moment
*W*	The weight matrix
*σ*	The sigmoid activation function
tan*h*	The tanh activation function
⊕	The vector concatenating function
*α*_it_	The normalized word weight of sentence *s*_i_
*s*_i_	The sentence vector
*u*_i_	The hidden representation of sentence vector *s*_i_
*α*_i_	The normalized sentence weight of sentence set *Se*_*pq*_
*v*_*s*_	The vector for *Se*_pq_
*μ* ^*t*^	The average value of input data
Σ^t^	The input variance
*G*, *b*	The bias constants
*f* (.)	The linear transformation function
Ζ	The regularization parameter
H(.)	The cross-entropy function

**Table 2:** Value of optimal parameters in the model.

Item	Value
Learning rate	0.001
Batch-size	50
Gru-size	128
Dropout	0.7
Bias constant in LN: *g*	0.001
Number of iterations	200
Embedding size	200
Layer number	4
Regularization parameter in LN: *ζ*	0.0001
Bias constant in LN: *t*	0.001

## Data Availability

The data used to support the findings of this study are available from the corresponding author upon request.
